# Evaluation of Midnight Salivary Cortisol as a Predictor Factor for Common Carotid Arteries Intima Media Thickness in Patients with Clinically Inapparent Adrenal Adenomas

**DOI:** 10.1155/2015/674734

**Published:** 2015-05-14

**Authors:** Giuseppe Reimondo, Barbara Allasino, Marcella Coletta, Anna Pia, Giulia Peraga, Barbara Zaggia, Chiara Massaglia, Piero Paccotti, Massimo Terzolo

**Affiliations:** Internal Medicine I, Department of Clinical and Biological Sciences, University of Turin, San Luigi Hospital, 10043 Orbassano, Italy

## Abstract

*Purpose*. The aim of the present study was to investigate the atherosclerotic vascular damage in a consecutive series of patients with AI and to correlate it with MSC.* Methods*. We studied 32 patients with AI matched with control subjects for age, sex, and cardiovascular risk factors. Either patients or control subjects underwent MSC measurement as outpatients and carotid arteries ultrasound (US) imaging studies.* Results*. The patients with AI had higher mean carotid artery IMT values and higher MSC levels than control subjects. In a multivariate analysis performed in AI age was the best predictor for IMT. We have stratified patients and control subjects by age (<60 yrs and ≥60 yrs). The patients showed significantly higher MSC levels than controls in both groups, whereas significantly higher IMT values were observed only in older subjects.* Conclusions*. Patients with AI have signs of accelerated atherosclerosis. Patients older than 60 years seem more susceptible to the possible detrimental effect of subclinical hypercortisolism on cardiovascular system. The MSC levels are not a strong predictor of the accelerated atherosclerosis, but they seem to indicate the subtle but not autonomous cortisol excess that may potentially raise the cardiovascular risk.

## 1. Introduction

Clinically inapparent adrenal masses are discovered serendipitously during diagnostic testing or treatment for unrelated disorders and are commonly known as adrenal incidentaloma (AI) [1–3]. Improvement in imaging techniques has resulted in the detection of an increasing number of AI, whose numbers count millions of people worldwide [3]. In 5–47% of cases depending on different diagnostic protocols, AI are associated with autonomous cortisol secretion that is no longer under control of pituitary feedback [4–8]. This particular endocrine disorder, which has been called subclinical Cushing's syndrome (SCS) [9] or subclinical autonomous glucocorticoid hypersecretion [2], is difficult to characterize because of a spectrum of variation from normality to autonomy of cortisol secretion and since the degree of cortisol excess is often minimal [2, 8, 10]. Notwithstanding the uncertainty regarding definition of SCS, many patients with the condition may be exposed to a chronic, low-grade cortisol excess. Results from different studies support the concept that this condition may confer an increased risk for metabolic disorders and cardiovascular events [11–16].

Previous reports suggested that patients with Cushing's syndrome had severe atherosclerotic damage, as indicated by reduced caliber, increased stiffness of carotid artery wall, and increased prevalence of atherosclerotic plaques [11, 12]. However, there are conflicting data on the possibility that also the low-grade cortisol excess characterizing SCS may cause vascular damage [13, 14].

The aim of the present study was to investigate the atherosclerotic vascular damage in a consecutive series of patients with AI and to correlate it with midnight salivary cortisol, as an outpatient measure of cortisol excess.

## 2. Patients and Methods

### 2.1. Patients

We studied a consecutive, prospective series of 32 patients with incidentally detected adrenocortical adenoma [13 men, 19 women; median age 61.5 years, range: 37–74] referred to our center from January 1, 2013, to February 28, 2014. In all cases, AI were detected by an abdominal CT scan done for unrelated diseases. The diagnosis of cortical adenoma rested on the following CT criteria: size less than 6.0 cm, regular shape with well-defined margins, homogenous texture, and hypodense content. An attenuation value of 10, or less, Hounsfield units on unenhanced CT scan was considered suggestive of an adrenal adenoma. The diagnosis of adenoma was confirmed by a repeat CT scan after 3–6 months showing no significant increase in mass size, or change in mass density, in any patient. The patients with confirmed diagnosis of hyperaldosteronism or pheochromocytoma were excluded.

We performed a 1 : 1 case-control analysis with 32 control subjects matched for age, sex, BMI, smoking status, blood pressure, glucose and lipid profile, and occurrence of previous cardiovascular events. They were collected from the medical staff and their relatives. All of the control subjects had previous imaging exclusion of adrenal masses.

Either patients or control subjects were studied as outpatients and they underwent careful clinical and history examination; none of them displayed specific signs or symptoms of hypercortisolism, such as weakness associated with proximal muscle wasting, skin atrophy, ecchymoses, or purple striae. Moreover, none of them was receiving any drug known to affect the hypothalamic-pituitary-adrenal axis or had current or previous history of alcohol abuse or major mood disorders. Either patients or controls had an apparently normal sleep-wake cycle.

Any subject with BMI greater than 30 kg/m^2^ was categorized as obese [17]. Any subject with systolic blood pressure greater than 140 mm Hg or diastolic blood pressure greater than 90 mm Hg or on antihypertensive treatment was categorized as hypertensive [18]. Visit blood pressure was the average of two seated measurements taken after five minutes of rest by a physician using standardized techniques. Diabetes mellitus was diagnosed if a subject was on insulin or hypoglycemic agents or when the subject's plasma glucose was greater than 7.0 mmol/L at fasting in at least two samples collected on different days [19]. Impaired fasting glucose was diagnosed when the subject's plasma glucose was between 6.1 and 7.0 mmol/L at fasting in at least two samples collected on different days [19]. Cardiovascular and cerebrovascular events were ascertained by reviewing the patient discharge summaries and source documents; complementary documentation was requested if necessary. Cardiovascular events were validated according to the principles used in randomized trials. The following definitions were used: (a) myocardial infarction was defined if there was hospitalization in the presence of 2 or more of the symptoms, typical chest pain, electrocardiographic changes, and increased cardiac enzyme concentrations, or by the presence of typical ECG changes without any previous acute symptoms (silent myocardial infarction); (b) angina pectoris was diagnosed if there were hospitalization and chest pain and documented electrocardiographic signs of coronary ischemia or if there was a need for coronary revascularization in the absence of acute myocardial infarction; (c) stroke was diagnosed if there was hospitalization for a neurologic deficit with symptoms continuing for more than 24 hours; (d) transient ischemic attack (TIA) was defined as a neurologic deficit lasting less than 24 hours. Smoking status was defined as active if patients were current or former smokers.

The institutional review board approved the study, and all subjects provided written, informed consent.

### 2.2. Methods

The patients with incidentally discovered adrenal adenoma underwent a specific endocrine evaluation as outpatients. It included (a) measurement of the 24-hour excretion of urinary free cortisol (UFC), (b) measurement of plasma ACTH at 8:00 a.m., and (c) overnight low-dose dexamethasone suppression test (1 mg, orally, at 11:00 p.m. with measurement of serum cortisol at 8:00 a.m. the following morning).

Either patients or control subjects underwent midnight salivary cortisol (MSC) measurement as outpatients. They were instructed to collect saliva samples at midnight. These were obtained twice and were kept in a refrigerator for up to 1 week before being transferred to our reference laboratory. Saliva was collected using Salivettes (Sarstedt, Newton, NC), a cotton device that is placed in the mouth for 2 min. Before saliva collection, patients were instructed to rinse their mouth without brushing their teeth (to avoid risk of gingival bleeding). Midnight samples were collected after 4-5 h of fasting (last food intake at 1900 or 2000 h). A mean of the two collected samples was calculated. The MSC was determined in our reference laboratory using commercially available kits (RIA, Radim S.p.A., Rome, Italy). The assay sensitivity was 0.5 nmol/L. The intra- and interassay variations were 3 and 9%, respectively. Coefficients of variation in the high and in the low range were, respectively, of 7 and 4%. Levels of salivary cortisol below 8.3 nmol/L were considered in the normal range. Concentrations were defined in a large series of healthy subjects.

Serum and urinary cortisol were measured using commercially available RIAs (Sorin Biomedica, Saluggia, Italy; Radim S.p.A., Pomezia, Rome, Italy; Diagnostic Product Corporation, Los Angeles, CA, USA). Intra-assay and interassay coefficients of variation were 6% and 11.5%, respectively. Plasma ACTH was measured by commercially available immunoradiometric assays (CIS Biointernational, Gif-sur-Yvette, France). Intra-assay coefficients of variation ranged from 2.1 to 5.3% and interassay coefficients of variation ranged from 3.1 to 8.9%, respectively; sensitivity ranged from 0.44 to 1.1 pmol/L.

SCS was considered in agreement with the recommendations of the AME Position Statement [20]: no specific clinical sign of cortisol excess and serum cortisol >138 nmol/L after 1 mg dexamethasone suppression test or serum cortisol between 50 nmol/L and 138 nmol/L with one additional alteration: UFC >414 nmol/24 h or ACTH <2.2 pmol/L.

Carotid arteries ultrasound imaging was performed in all subjects by echo-Doppler ultrasonography (US), carried out with a Vingmed Sound CMF 725 (Horten, Norway) using a 7.5 MHz annular phased array transducer. Right and left carotid arteries were scanned longitudinally, 2.5 cm proximal to the bifurcation. The pictures were stored on magnetic media and analyzed later. US imaging studies were performed by the same operator (B.A.), who was blinded to laboratory assessment. Each measurement was repeated three times, and the mean was determined. Wall thickness of both carotid arteries was investigated by measuring the intima media thickness. In all subjects, the presence, location, and size of plaques were also evaluated at the levels of common, internal, and external carotid arteries.

### 2.3. Statistical Analysis

Rates and proportions were calculated for categorical data and means and standard deviations for continuous data. Normality of data was assessed by the Kolmogorov-Smirnov test. For continuous variables, differences were analyzed by means of the two-tailed Student's *t*-test when data were normally distributed and by using the Mann-Whitney *U* test for nonparametric data. For categorical variables, differences were analysed by means of the *χ* ^2^ test with Yates correction. Levels of statistical significance were set at *p* < 0.05. All analyses were performed using the SPSS software package version 21 (IBM).

## 3. Results

The characteristics of the patients with AI are reported in Table 1. Eleven patients (34.4%) qualified for SCS and eight patients (25%) had MSC >8.3 nmol/L, whereas in none of the controls elevated MSC levels were observed. Eighteen patients (56.2%) had hypertension and 16 patients (50%) had hyperglycemia (Table 1).

The whole group of patients with AI had higher IMT values than control subjects (left IMT: 0.069 ± 0.017 cm versus 0.060 ± 0.010 cm, *p* = 0.013; right IMT: 0.072 ± 0.018 cm versus 0.061 ± 0.010 cm, *p* = 0.005). Four AI patients had IMT values >0.9 mm, while all of the controls had IMT in the normal range. Well-defined carotid wall plaques were detected in six patients (18.7%) and only in one control subject (3.1%). Two patients had a plaque localized at the level of the left carotid bifurcation with maximum diameters of six mm and seven mm, respectively, whereas four patients had a plaque at the level of the right internal carotid artery with a maximal diameter ranging from six mm to eight mm. A well-defined carotid wall plaque with maximal diameter of nine mm was detected in one control at level of the right carotid artery bifurcation. The same figure was observed when we considered the two groups of patients with SCS or nonsecreting AI compared to the matched controls (Table 2(a)), while the patients with SCS did not present any significant difference for either IMT values or clinical parameters in comparison with patients with nonsecreting AI (Table 2(b)).

The patients with AI had higher midnight salivary cortisol levels than control subjects (5.90 ± 4.17 nmol/L versus 1.90 ± 0.77 nmol/L, *p* < 0.0001). When patients were classified for MSC levels (>8.3 nmol/L, Group A, and ≤8.3 nmol/L, Group B) we have not observed any significant difference for either IMT values or clinical parameters (Table 3(a)). However, both groups had higher IMT values than the matched controls (Table 3(b)).

In univariate analysis we have found a correlation at the limit of the statistical significance between IMT and hypertension (*r* = 0.378, *p* = 0.062), smoking status (*p* = 0.372, *p* = 0.067), or cortisol levels after 1 mg DST (*r* = 0.363, *p* = 0.074). A significant correlation has been found with age (*r* = 0.478, *p* = 0.016), while we have not found any correlation between IMT and obesity, hyperglycemia, MSC, UFC, or ACTH.

In a multivariate analysis performed in patients with AI that included hypertension, smoking status, age, and cortisol levels after 1 mg DST, age was the best predictor for both left IMT and right IMT (*r* ^2^ = 0.466, *p* = 0.001). Therefore, we have stratified patients and control subjects by age defining 2 groups (<60 years and ≥60 years) which represent the mean age of the series. The patients showed significantly higher MSC levels than controls in both age groups, whereas significantly higher IMT values were observed only in older subjects (Table 4).

## 4. Discussion

The present data demonstrate that patients with AI and of 60 years or more have higher carotid artery IMT values than control subjects matched for age, sex, BMI, smoking status, blood pressure, glycemic state, lipid profile, and occurrence of previous cardiovascular events. Moreover, the overall group of patients shows higher MSC levels in comparison with control subjects. However, MSC was not a predictor of increased IMT.

The link between cortisol and atherosclerosis is suggested by fragments of information indicating that prolonged corticosteroid therapy accelerates the development of atherosclerosis. In animal experiments, ACTH and cortisone were shown to produce vascular injury and to enhance experimentally induced atherosclerosis [21, 22]. In humans, the use of glucocorticoids caused significant changes in vascular connective tissue [23–25]. In addition, a cause and effect relationship between corticosteroid treatment and premature atherosclerosis was hypothesized in patients with rheumatoid arthritis or systemic lupus erythematosus chronically treated with glucocorticoids [26, 27]. Furthermore, a significant association was found between coronary artery disease and serum cortisol concentrations in patients subjected to coronary angiography [24]. In patients with endogenous hypercortisolism, the presence of atherosclerotic plaques or preatherosclerotic lesions at the level of coronary or carotid arteries has been previously demonstrated [11, 12, 28].

Several data from cross-sectional studies point to an association between clinically inapparent cortisol excess and some manifestations of the metabolic syndrome (arterial hypertension, hyperglycemia, and overweight) and predisposition to thrombosis and cardiovascular disease in patients with adrenal incidentaloma [4, 13–15, 29–31].

Carotid IMT is generally recognized as a marker of early atherosclerosis. In fact, ultrasound examination of the carotid arteries with measurement of the IMT and detection of plaques has repeatedly been shown to predict occurrence of both stroke and myocardial infarction. The relation between carotid artery IMT and cardiovascular events is continuous, but a threshold ≥0.9 mm can be considered as a conservative estimate of significant alteration [32].

In 2002 Tauchmanovà et al. [14] reported that carotid IMT was significantly increased in patients with subclinical cortisol excess compared with that in controls, suggesting a higher prevalence of systemic atherosclerosis, a concept supported by the increased frequency of atherosclerotic plaques. However, carotid IMT was not correlated with any hormonal parameters. More recent studies have confirmed, in different settings, increased IMT in patients with AI, either with or without subclinical cortisol excess [29, 33, 34]. In these papers, a more robust link between IMT and cortisol levels has been demonstrated, in particular with morning serum cortisol [29, 33, 34].

MSC is already an established test to screen patients with suspected overt Cushing's syndrome [35]. Nonetheless, in patients with AI evaluation of MSC was powerless to distinguish between nonsecreting adenoma and subclinical cortisol excess [36, 37]. Thus, none of the studies have evaluated MSC as a possible marker of increased IMT. MSC has the obvious advantage of being more practical; thus it is tempting to see if it can predict cardiovascular risk profile. Only one paper showed a correlation between IMT and morning salivary cortisol in healthy subjects [38]. In patients with AI, we have previously demonstrated that elevated midnight serum cortisol was associated with the elderly, greater fasting glucose, and systolic blood pressure [16].

Our findings on IMT are in agreement with previous studies confirming increased IMT and more wall plaque in AI (secreting and nonsecreting) compared to controls. Moreover, we have demonstrated that the whole group of AI patients presents higher MSC levels than controls, and 25% of them had MSC levels higher than the upper limit of normality. However, we have not confirmed a correlation between IMT and hormonal and metabolic factors, whereas the only predictor was age. We acknowledge the limit of a reduced number of patients that may have hampered the demonstration of a correlation between IMT and hormonal variables. Moreover, the hormonal evaluations of patients with AI remain controversial, since we use parameters that are not representative of the possible persistent exposure to cortisol excess. Thus, also patients with AI and formally normal hormonal value should have metabolic alterations and an increased cardiovascular risk. On the other hand the strength has been to have designed our study to evaluate IMT in patients with AI carefully matched to control subjects for age and known cardiovascular risk factors. Nonetheless, our data support the hypothesis that patients with AI may present a worsened cardiovascular risk profile when compared to the general population, as a possible consequence of being exposed to a chronic cortisol excess, albeit of minimal intensity. Patients with AI showed higher MSC than controls, as an additional marker of an altered HPA axis. Additionally, our data point out the importance of assessing IMT in patients older than 60 years.

In conclusion, the present findings confirm that patients with AI show signs of accelerated atherosclerosis compared to matched controls. Patients older than 60 years seem more compromised. Levels of MSC are not a strong predictor of accelerated atherosclerosis, although further studies are needed to fully evaluate whether MSC may be exploited as a marker of subclinical cortisol excess that may potentially raise cardiovascular risk.

## Figures and Tables

**Table 1:** Demographic, anthropometric, and hormonal characteristics of the patients with AI.

Patients	Age	Sex	BMI	Hypertension	IGT/diabetes	Mass size	MSC	UFC	ACTH	Cortisol after 1 mg DST	SCS	Left IMT	Right IMT
	(years)	(M/F)	(kg/m^2^)	(Y/N)	(Y/N)	(cm)	(nmol/L)	(nmol/24 h)	(pmol/L)	(nmol/L)	(Y/N)	(cm)	(cm)
#1	58	F	23.1	Y	N	2.2	4.7	118.6	2.4	179.4	Y	0.07	0.07
#2	56	F	23.5	N	N	3.5	2.7	126.9	4.6	66.2	N	0.08	0.08
#3	57	F	24.0	Y	N	2.4	5.8	750.7	2.4	44.1	Y	0.05	0.05
#4	56	M	27.5	N	N	3.0	2.7	540.9	9.0	55.2	Y	0.06	0.08
#5	58	F	24.4	Y	Y	2.0	22.1	110.4	2.2	44.1	N	0.08	0.08
#6	44	F	37.7	Y	Y	2.0	8.5	162.8	18.9	27.6	N	0.05	0.06
#7	53	F	21.6	N	N	2.5	7.7	52.4	2.3	74.5	N	0.06	0.08
#8	37	F	27.6	N	N	1.9	8.5	836.2	5.7	113.1	Y	0.03	0.03
#9	56	F	34.0	Y	Y	2.4	8.5	237.3	1.7	77.2	Y	0.08	0.07
#10	53	F	20.7	Y	N	3.3	2.7	494.1	1.6	41.4	N	0.06	0.06
#11	43	F	20.7	N	Y	3.5	2.7	460.9	3.5	46.9	N	0.06	0.06
#12	60	F	36.0	N	N	1.5	2.7	242.8	4.6	63.4	N	0.07	0.08
#13	43	M	26.6	N	N	3.0	5.5	184.9	3.3	41.4	N	0.07	0.08
#14	58	F	36.9	N	Y	2.7	2.7	314.6	4.6	184.9	Y	0.05	0.05
#15	46	F	21.0	N	N	2.0	2.7	474.7	3.5	41.4	N	0.05	0.05
#16	71	M	24.5	N	N	2.0	2.7	411.2	3.4	66.2	N	0.08	0.08
#17	70	F	23.4	Y	N	4.0	7.4	322.9	1.5	55.2	Y	0.08	0.08
#18	63	M	28.0	Y	N	2.0	6.6	673.4	1.1	41.4	N	0.06	0.05
#19	67	M	27.7	Y	N	2.5	2.7	298.1	4.1	82.8	N	0.07	0.07
#20	72	M	29.6	Y	Y	3.0	2.7	146.2	4.6	77.2	N	0.11	0.12
#21	64	F	20.3	N	Y	3.0	3.8	140.7	4.8	71.7	N	0.06	0.07
#22	70	F	19.0	Y	N	3.0	9.9	146.2	1.1	146.8	Y	0.09	0.10
#23	62	M	29.4	Y	Y	2.0	2.7	342.2	1.1	44.1	N	0.09	0.08
#24	64	F	26.9	Y	Y	2.0	4.7	226.3	0.7	113.1	Y	0.08	0.07
#25	64	M	27.3	Y	Y	3.7	6.9	447.1	3.3	110.4	Y	0.06	0.07
#26	62	M	26.8	Y	Y	3.0	2.7	113.1	2.6	66.2	N	0.08	0.08
#27	62	M	27.2	N	N	3.5	2.7	419.5	3.3	55.2	Y	0.06	0.07
#28	74	M	29.7	Y	Y	6.0	2.7	171.1	3.3	124.2	N	0.08	0.08
#29	70	F	27.9	N	Y	2.5	13.2	794.8	2.8	38.6	N	0.05	0.05
#30	70	M	28.1	N	Y	2.7	11.0	126.9	4.6	27.6	N	0.05	0.05
#31	61	F	28.0	Y	Y	3.0	8.5	187.6	2.2	38.6	N	0.10	0.10
#32	68	M	31.8	Y	Y	2.5	8.0	187.6	1.1	129.7	Y	0.09	0.10

SCS = subclinical Cushing syndrome, AI = adrenal incidentaloma, IMT = intima media thickness, BMI = body mass index, IGT = impaired glucose tolerance, Y = yes, N = no, MSC = midnight salivary cortisol, UFC = urinary free cortisol, and ACTH = adrenocorticotropin hormone.

**(a):** 

	*N*	Mean	SD	*p* value
Left IMT (cm)				
SCS	11	0.068	0.018	0.01
Controls	11	0.060	0.007	
Right IMT (cm)				
SCS	11	0.071	0.019	0.04
Controls	11	0.063	0.009	
Left IMT (cm)				
Nonsecreting	21	0.069	0.017	0.03
Controls	21	0.059	0.011	
Right IMT (cm)				
Nonsecreting	21	0.071	0.018	0.01
Controls	21	0.059	0.011	

**(b):** 

	*N*	Mean	SD	%	*p* value
Left IMT (cm)					
SCS	11	0.068	0.018		NS
Nonsecreting	21	0.069	0.017		
Right IMT (cm)					
SCS	11	0.071	0.019		NS
Nonsecreting	21	0.071	0.018		
Age (yrs)					
SCS	11	60.2	9.3		NS
Nonsecreting	21	59.4	9.7		
BMI (kg/m^2^)					
SCS	11	27.7	5.1		NS
Nonsecreting	21	26.4	4.6		
Mass size (cm)					
SCS	11	2.8	0.7		NS
Nonsecreting	21	2.7	0.9		
Hypertension (%)					
SCS	11			63.6	NS
Nonsecreting	21			52.4	
IGT/diabetes (%)					
SCS	11			45.4	NS
Nonsecreting	21			47.6	

SCS = subclinical Cushing syndrome, AI = adrenal incidentaloma, IMT = intima media thickness, BMI = body mass index, IGT = impaired glucose tolerance, *N* = numbers of patients, SD = standard deviations, and NS = not significant.

**(a):** 

	*N*	Mean	SD	%	*p* value
Left IMT (cm)					
Group A	8	0.068	0.024		NS
Group B	24	0.070	0.014		
Right IMT (cm)					
Group A	8	0.069	0.024		NS
Group B	24	0.071	0.016		
Age (yrs)					
Group A	8	58.2	12.4		NS
Group B	24	60.2	8.5		
BMI (kg/m^2^)					
Group A	8	28.3	5.6		NS
Group B	24	26.4	4.4		
Mass size (cm)					
Group A	8	2.5	0.4		NS
Group B	24	2.8	0.9		
Hypertension (%)					
Group A	8			62.5	NS
Group B	24			54.2	
IGT/diabetes (%)					
Group A	8			75.0	NS
Group B	24			71.4	

**(b):** 

	*N*	Mean	SD	*p* value
Left IMT (cm)				
Group A	8	0.066	0.024	0.01
Controls	8	0.0588	0.009	
Right IMT (cm)				
Group A	8	0.067	0.024	0.03
Controls	8	0.057	0.011	
Left IMT (cm)				
Group B	24	0.070	0.014	0.01
Controls	24	0.060	0.010	
Right IMT (cm)				
Group B	24	0.073	0.016	0.006
Controls	24	0.062	0.010	

IMT = intima media thickness, BMI = body mass index, IGT = impaired glucose tolerance, *N* = numbers of patients, SD = standard deviations, and NS = not significant.

**Table 4:** Comparison between patients and controls stratified by age.

	Left IMT (cm)			Right IMT (cm)			Midnight salivary cortisol (nmol/L)		
	Patients (#16)	Controls (#16)	*p*	Patients (#16)	Controls (#16)	*p*	Patients (#16)	Controls (#16)	*p*
Age ≥ 60 yrs	0.075 ± 0.017	0.060 ± 0.012	0.005	0.077 ± 0.019	0.060 ± 0.012	0.006	5.85 ± 3.39	2.12 ± 0.66	0.001
Age < 60 yrs	0.061 ± 0.014	0.060 ± 0.008	NS	0.065 ± 0.015	0.060 ± 0.009	NS	5.98 ± 4.99	1.68 ± 0.77	0.002
